# Collodion and Asbestos in Toothache

**Published:** 1849-03

**Authors:** 


*Collodion and Asbestos for Toothache.*—Mr. Robinson, a distinguished dentist of London, says that he has frequently applied collodion in severe cases of toothache arising from exposure of the nerve. The method he adopts, is to make the patient first wash out his mouth in warm water, in which a few grains of bicarbonate of soda has been dissolved. He then removes from the cavity any foreign substance likely to cause irritation. After drying the cavity, he drops from a point, the collodion, to which has been added a few grains of morphia; after which, he fills the cavity with asbestos, and saturates with the collodion. Lastly, over this he places a pledget of bibulous paper. In a few seconds the whole becomes solidified, and forms an excellent nonconductor of heat or cold to the exposed nerve. By occasionally renewing this, he has been enabled to effect a more durable stopping than with gold.—*Medical Times.*—*Ibid.*

